# Transcriptional Analysis of Carotenoids Accumulation and Metabolism in a Pink-Fleshed Lemon Mutant

**DOI:** 10.3390/genes11111294

**Published:** 2020-10-30

**Authors:** Giuseppe Lana, Jaime Zacarias-Garcia, Gaetano Distefano, Alessandra Gentile, María J. Rodrigo, Lorenzo Zacarias

**Affiliations:** 1 Department of Agriculture, Food and Environment, University of Catania, 95123 Catania, Italy; giuseppe.lana51@gmail.com (G.L.); distefag@unict.it (G.D.); gentilea@unict.it (A.G.); 2 Food Biotechnology Department, Instituto de Agroquímica y Tecnología de Alimentos, Consejo Superior de Investigaciones Científicas (IATA-CSIC), Paterna, 46980 Valencia, Spain; jaizagar@iata.csic.es (J.Z.-G.); mjrodrigo@iata.csic.es (M.J.R.)

**Keywords:** carotenoids, citrus, gene expression, fruit quality, *Citrus limon*, lycopene, pigments

## Abstract

Pink lemon is a spontaneous bud mutation of lemon (*Citrus limon*, L. Burm. f) characterized by the production of pink-fleshed fruits due to an unusual accumulation of lycopene. To elucidate the genetic determinism of the altered pigmentation, comparative carotenoid profiling and transcriptional analysis of both the genes involved in carotenoid precursors and metabolism, and the proteins related to carotenoid-sequestering structures were performed in pink-fleshed lemon and its wild-type. The carotenoid profile of pink lemon pulp is characterized by an increased accumulation of linear carotenoids, such as lycopene, phytoene and phytofluene, from the early stages of development, reaching their maximum in mature green fruits. The distinctive phenotype of pink lemon is associated with an up-regulation and down-regulation of the genes upstream and downstream the lycopene cyclase, respectively. In particular, 9-cis epoxycarotenoid dioxygenase genes were overexpressed in pink lemon compared with the wild-type, suggesting an altered regulation of abscisic acid biosynthesis. Similarly, during early development of the fruits, genes of the carotenoid-associated proteins heat shock protein 21, fibrillin 1 and 2 and orange gene were overexpressed in the pulp of the pink-fleshed lemon compared to the wild-type, indicating its increased capacity for sequestration, stabilization or accumulation of carotenes. Altogether, the results highlighted significant differences at the transcriptomic level between the pink-fleshed lemon and its wild-type, in terms of carotenoid metabolism and the capacity of stabilization in storage structures between the two accessions. Such changes may be either responsible for the altered carotenoid accumulation or in contrast, a metabolic consequence.

## 1. Introduction

Carotenoids are isoprenoids-derived molecules that carry out essential functions in plant cells; they are part of the photosynthetic system and participate in light capture. Carotenoids play an important role in photo-protection, increasing tolerance to light and heat stresses while preventing membranes from lipid peroxidation. These pigments are also precursors of important phytohormones such as abscisic acid (ABA) and strigolactones; carotenoids constitute the substrates for the formation of apocarotenoids-derived volatiles. In addition, they are involved in plant–animal interactions [1].

Carotenoids are not only responsible for the attractive color of flowers, fruits and other organs in many plant species, but they are also known for their benefits to human health. These properties are mainly due to the antioxidant activity and the fact that α- and β-carotene and β-cryptoxanthin are precursors of vitamin A, an essential dietary component [2]. Recent studies highlighted that a regular intake of carotenoids has a positive effect on human health by preventing neurodegenerative, cardiovascular and aging-related diseases, as well as reducing cancer risk [2,3,4].

Citrus fruit pigmentation is characterized by wide variability, with the peel and pulp color ranging from the pale yellow of lemons, pummelos and grapefruits, to the light and deep orange of oranges and mandarins, respectively, to the reddish shades of red grapefruits and some orange mutants [5,6]. This variability in pigmentation is mainly due to the differences in carotenoid accumulation and composition, which is responsible for the species-specific color according to the ripening stages [6,7]. Citrus fruits are consumed worldwide for their organoleptic characteristics as well as their nutraceutical value [8]. In light of this, unravelling the modifications that occur in mutant phenotypes is a prerequisite to identify new genetic markers associated with improved commercial and nutritional traits. Indeed, molecular markers are a fundamental tool for breeders to reduce the amount of time and money required to develop a new cultivar through traditional breeding.

In plants, carotenoids are generally formed by the condensation of eight C5 isoprenoid units that form a C40 polyene backbone containing a variable number of conjugated double bonds. This particular chemical structure gives carotenoids the capacity to absorb visible light at different wavelengths. Carotenoids are classified as carotenes and xanthophylls, the former composed exclusively of carbon and hydrogen atoms, the latter containing at least one oxygenated group [9,10]. Over recent decades, several genes encoding for enzymes involved in the main steps of carotenoid biosynthesis pathway have been isolated and their molecular and biochemical regulation has been clarified [10,11]. Moreover, other processes related to the storage of carotenoids in chromoplasts and how they are catabolized by a family of enzymes known as carotenoid cleavage dioxygenases (CCDs) have also been addressed [12,13]. The expression of a large number of carotenoid biosynthetic genes has been studied in the peel and pulp of many citrus varieties during the ripening process [7,14].

**Figure 1:**
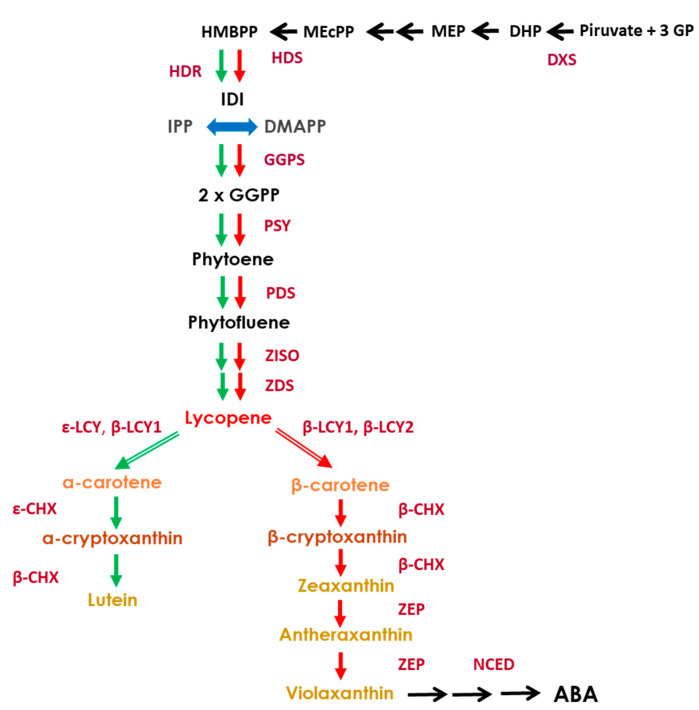
Schematic representation of carotenoid biosynthesis in citrus fruits, indicating main enzymes and genes of the pathway. 1-deoxy-D-xylulose-5-phosphate synthase (*DXS*), hydroxymethylbutenyl diphosphate synthase (*HDS*) and reductase (*HDR*), geranylgeranyl diphosphate synthase (*GGPSS*), phytoene synthase (*PSY*), phytoene desaturase (*PDS*), ζ-carotene isomerase (*ZISO*), ζ-carotene desaturase (*ZDS*), ε-lycopene cyclase (*ε-LCY*), β-lycopene cyclase (*β-LCY1*/*2*), ε-carotene hydroxylase (*ε-CHX*), β-carotene hydroxylase (*β-CHX*), zeaxanthin epoxidase (*ZEP*) and 9-cis-epoxy-carotenoid dioxygenase (*NCED*). Green and red arrows represent carotenoid biosynthesis flux in green and mature tissues, respectively.

The initial substrate for carotenoid biosynthesis, geranylgeranyl diphosphate (C20, GGPP), is produced by the condensation of one dimethylallyl diphosphate (DMAP) and three isopentenyl diphosphate (IPP) molecules (Figure 1). The synthesis of these precursors takes place through the so-called methylerythritol 4-phosphate (MEP) pathway and involves the participation of several enzymes, such as 1-deoxy-D-xylulose-5-phosphate synthase (DXS), which is located upstream, and hydroxymethylbutenyl diphosphate synthase (HDS) and reductase (HDR), which are located downstream of the pathway. At the end of the MEP pathway, the formation of GGPP is catalyzed by the geranyl geranyl pyrophosphate synthase (GGPPS) enzyme which is coded by a multigene family [1]. During the first two steps of carotenoid formation, phytoene synthase (*PSY*) and phytoene desaturase (*PDS*) catalyzes the head-to-head condensation of two molecules of GGPP to form the colorless phytoene (C40) and phytofluene. Subsequently, desaturation and isomerization by ζ-carotene desaturase (*ZDS*) and ζ-carotene isomerase (*Z-ISO*) produce lycopene, through the intermediates ζ-carotene and neurosporene. At this point, the pathway splits into two branches. Lycopene ε-cyclase (*ε -LCY*) and lycopene β-cyclase (*β-LCY*) are responsible for the addition of one or two β-ionone rings producing δ-carotene and β-carotene, respectively. Subsequently, *β-LCY* introduces a second β-ionone ring on δ-carotene to produce α-carotene [7,15]. Two subfamilies of β-lycopene cyclases have been identified in citrus fruits—*β-LCY1* and *β-LCY2*. The first of these genes shows a constant expression during the ripening process and is expressed in a large variety of organs and tissues, while the second is chromoplast-specific and is typically expressed in fruit tissues; it is highly up-regulated during the fruit maturation phase [16]. Two different alleles of *β-LCY2* have been isolated: *β-LCY2a*, and *β-LCY2b.* Studies carried out on both variants revealed a differential tissue and temporal expression, as well as a different enzymatic efficiency to convert lycopene into β-carotene [17,18]. δ-carotene is converted into *β*-carotene and then into lutein by β-carotene hydroxylase, while, β-carotene is hydroxylated to β-cryptoxanthin and zeaxanthin by β-carotene hydroxylase (*β-CHX*) [19]. In citrus fruits, these two last carotenoids can be catabolized to C30-apocarotenoids by a class of enzymes generally recognized as carotenoids cleavage dioxygenases (*CCDs*) [5,20,21]. Zeaxanthin epoxidase (*ZEP*) adds to antheraxanthin and, subsequently, to violaxanthin epoxy groups resulting in neoxanthin formation. The last reaction of the pathway is catalyzed by neoxanthin synthase (*NSY*), which turns violaxanthin into neoxanthin. The 9-cis-isomers of these last two xanthophylls are then utilized as substrates by 9-cis epoxycarotenoid dioxygenase (*NCED*) to produce ABA [22,23].

Carotenoid synthesis takes place concurrently with the differentiation of chromoplasts, leading to the development of diverse sink structures organized to store the newly produced carotenoid [24]. The ultrastructural changes that occur during this phase involve several proteins. Among them, the most important are the small heat shock proteins (sHSPs), fibrillins (FIBs or PAPs) and orange gene protein (*OR*). *HSP21* transcript level has been correlated with carotenoid accumulation in tomato fruit [25]. Fibrillins play a structural role in fibrils, organizing carotenoids in lipoprotein complexes [26]. Several studies have demonstrated that OR protein promotes PSY activity and chromoplast biogenesis, which leads to enhanced carotenoid accumulation [24,27,28].

The large variety of pigmentation showed by both rind and flesh of mature citrus fruits is strictly related to the differences in the total amount and composition of carotenoids typical of each species and cultivar [7,15]. In the case of ordinary lemon (*Citrus limon*), the light-yellow coloration is due to a very low accumulation of carotenoids [9,29]. Comparative transcriptomic analysis has highlighted a reduced expression of most of the carotenoids biosynthetic genes in both flavedo and juice sacs of lemon fruits compared to those found in oranges and mandarins [29].

Accumulation of lycopene in *Citrus* is relatively uncommon and characterizes just a few varieties and mutants of pummelo, grapefruit and sweet orange. Despite the extensive efforts to investigate carotenoid biosynthesis and metabolism in several red-fleshed citrus mutants, the molecular basis of lycopene accumulation has not been completely elucidated yet [7,15]. In the case of Cara Cara orange mutant, it has been proposed that the red pigmentation is amenable to an enhanced flow of carotenoids precursors through the MEP pathway [30,31]. In addition, it has been found that alterations in the expression of the two alleles of *β-LCY2* might lead to the accumulation of lycopene [17,32,33,34,35]. Lycopene cyclase activity is a rate-limiting step in the biosynthesis of carotenoids, then a partial blockage in the conversion of lycopene to β-carotene may increase the accumulation of lycopene and repress the production of downstream metabolites like xanthophylls [17,18,32,33]. A comparison between white and red pummelos indicated that lycopene accumulation is associated with a reduced expression of genes encoding for enzymes which operate downstream lycopene production [14,36,37,38], reinforcing the hypothesis that a reduction of activity of lycopene cyclase might contribute to the onset of a bottleneck along the carotenoid pathway.

**Figure 2:**
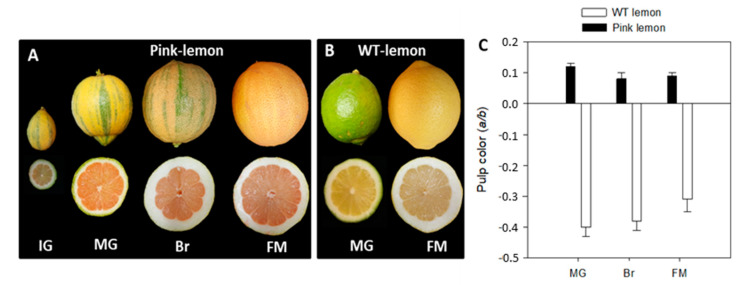
External and internal appearance of pink lemon (PL) (**A**) and wild-type (WT) (**B**) lemon fruit during development and maturation. Immature green (IG), mature green (MG), breaker (BR) and fully mature (FM) stages. Changes in color (a/b Hunter) of the pulp of PL and WT (**C**) during fruit maturation. Data are the mean ± SD of at least 10 fruits.

A pink-fleshed lemon was described in 1932 in California as a spontaneous bud mutation of Eureka lemon. The peel of pink lemon is variegated with green stripes, which turn yellow when mature, while the yellow section becomes light-pink (Figure 2A). The pulp has a light-pink coloration due to lycopene accumulation with few seeds and a sour taste when fully mature [39]. Although the mutant has been known for a long time and is commercially available in specialized markets, no information is available about the transcriptomic and metabolic changes behind the pink pigmentation.

The aim of the present work was to carry out a comparative analysis of carotenoids biosynthesis between the pulp of the pink-fleshed lemon and its wild type (WT), in order to elucidate the metabolic and molecular changes at the basis of the pigmentation of the red-fleshed mutant. To this end, the identification and quantification of carotenoids were performed using a HPLC-PDA technique, while the regulation of the genes involved in carotenoids biosynthesis and the production of proteins related to carotenoid-sequestering structures were detected through qPCR. An increased understanding of the genetic determinism of the pink-fleshed lemon phenotype could be of great interest to identify candidate genes for the development of molecular markers to be employed in fruit quality breeding programmes.

## 2. Materials and Methods

### 2.1. Plant Material

Fruits of pink lemon (PL) and Fino (*Citrus limon*, cv. Fino), referred to as wild type (WT) lemon, were harvested from adult trees grafted on Citrange carrizo (*Poncirus trifoliate* L. Raf × *Citrus sinensis* L. Osb) rootstocks cultivated at The Citrus Germplasm Bank (Instituto Valenciano de Investigaciones Agrarias, Moncada, Valencia, Spain) and were subjected to standard cultural practices. Samples were collected at four developmental stages: Immature green (IG), mature green (MG), breaker (BR) and fully mature (FM) (Figure 2). Trees of both genotypes were located in the same orchards and samples of each genotype were collected at the same time. Fruits were quickly delivered to the laboratory, where the pulp was separated from flavedo, frozen in liquid nitrogen, ground to a fine powder and stored at −80 °C until analysis. The color of the pulp was measured using a CR-400 Minolta chromameter (Konica Minolta, Tokyo, Japan) on three different locations around the equatorial plan of the fruits. The Hunter parameters *a* (negative to positive, from green to red) and *b* (negative to positive, from blue to yellow) were measured, and the color was expressed as the *a*/*b* Hunter ratio, a color index that has been widely used for color measurement in citrus fruit [40]. Data regarding the color index for each cultivar are the means ± SD of at least 10 fruits. Fruits were harvested and color was determined in two consecutive crop seasons.

### 2.2. Carotenoid Extraction and Quantification by HPLC-PDA

Carotenoids were extracted from frozen flesh following the protocol described by Rodrigo et al. [41]. Extracts were dried and kept at −20 °C until further analysis. Each sample was extracted in triplicate and results were expressed as mean ± SD. In order to prevent photodegradation, isomerizations and structural changes of carotenoids all the operations were carried out on ice under dim light.

Individual carotenoid analysis of each sample was carried out by HPLC-PDA, as described by Lado et al. [42] and Rodrigo et al. [41]. Carotenoids were identified by their absorption, fine spectra and retention time. Then, they were quantified integrating each one of them at its corresponding maximum absorbance wavelength and using the corresponding calibration curves, as reported by Rodrigo et al. [41].

### 2.3. Gene Expression Analysis by Quantitative Real-Time PCR

RNA isolation, cDNA synthesis and gene expression analyses were performed as described by Rodrigo et al. [43], and subsequently treated with DNA free, DNase treatment and removal (Ambion, Madrid, Spain) to eliminate any residual trace of DNA. Total RNA was quantified in a NanoDrop ND-1000 spectrophotometer (Thermo Fisher Scientific, Madrid, Spain) and the absence of DNA was checked by gel electrophoresis.

Briefly, 2 μg of total RNA was reverse transcribed using the SuperScript III Reverse Transcriptase (Invitrogen, Madrid, Spain) following the manufacturer’s instructions. Quantitative real-time polymerase chain reaction (qRT-PCR) was performed on a LightCycler 480 instrument (Roche, Madrid, Spain) using the LightCycler 480 SYBRGreen I Master kit (Roche). The primers employed for the amplification of each gene are listed in Table S1. Then, 20 ng of cDNA was used for each amplification reaction in a total volume of 10 μL. The cycling protocol consisted of 10 min at 95 °C for pre-incubation, followed by 35 cycles for 10 s at 95 °C for denaturation, 10 s at 59 °C for annealing and 10 s at 72 °C for extension. Fluorescence data were acquired at the end of extension phase and reactions specificity was checked by post-amplification dissociation curve. For expression measurements, we used the LightCycler 480 Software release 1.5.0, version 1.5.0.39 (Roche, Madrid, Spain), and calculated expression levels relative to the values of a reference sample using the Relative Expression Software Tool [44]. Actin gene expression was chosen to normalize raw Cp’s based on a previous selection of reference genes [45]. The results were the average of three independent sample replicates.

### 2.4. Statistical Analysis

The outputs of both HPLC-PDA and qPCR analysis were processed using the R software (R Development Core Team, 2016; R Foundation for Statistical Computing, Vienna, Austria). An ANOVA test was employed to determine significant differences (*p* value < 0.01) between pink lemon and its wild type. A Shapiro–Wilk test was performed before the ANOVA test.

## 3. Results

### 3.1. Phenotypic Characteristics of the Pink Lemon Fruit

Pink-fleshed lemon trees are characterized by variegated leaves and fruits, both of which can be characterized by their green and white sectors which are variable in shape and size. The typical green stripes were unevenly distributed on the fruit skin and even if they were evident from early developmental stages, during the maturation process, their color changed to the characteristic yellow, while the white areas turned light pink (Figure 2A). The red tone of the pulp was evident in the IG phase and increased in intensity with maturation, reaching an intense red color at the MG phase. The reddish coloration of the pulp at early stages moved to clearer shades, probably due to the dilution effect caused by the substantial growth of the pulp along the maturation process (Figure 2A). Remarkable differences in pulp color (determined as *a/b* Hunter ratio) were found between the two genotypes (Figure 2C). The color of pink lemon (PL) pulp assumed positive values at all developmental stages, although they slightly decreased as maturation progressed. By contrast, the color of wild-type (WT) lemon pulp assumed negative values typical of a light-yellow tone and it showed an increasing trend over the course of maturation (Figure 2B).

### 3.2. Carotenoids Content and Composition in Pink Lemon Fruit

Carotenoids content and composition were analyzed in the pulp of PL and WT fruits at four developmental stages, going from immature green to full maturity, as outlined in the Material and Methods section (Table 1). HPLC-PDA analysis facilitated the detection and quantification of eleven carotenoids. Carotenoids content and composition in PL fruits were markedly different from WT (Table 1; Figure S1) at all the four stages analyzed. Total carotenoids content was much higher in PL fruits than in WT fruits, differing by two to three orders of magnitude (Table 1). Total carotenoids content was very low (<0.4 µg/g FW) in WT fruits at all developmental stages, while in PL fruits the total carotenoids reached a maximum at MG (53.3 µg/g FW) and declined afterwards. The colorless phytoene and phytofluene were the major carotenes detected in PL flesh, accounting for 82–86% and 11–16% of total carotenoids, respectively, while these carotenes were only detected in traces or at extremely low levels in the pulp of WT fruits. In addition to phytoene and phytofluene, low amounts of lycopene, neurosporene, ζ- and δ-carotene were detected in the pulp of PL. In the pulp of mature WT fruits, only low levels of β-cryptoxanthin and traces of other carotenoids were detected (Table 1).

### 3.3. Expression of the Genes Involved in the Biochemical Pathway of Carotenoids

The expression levels of eleven genes related to carotenoids biosynthesis were tested through a qRT-PCR assay to explore the possible causal relationship between increased carotenoid accumulation in PL and the transcripts abundance of such candidate genes.

**Figure 3:**
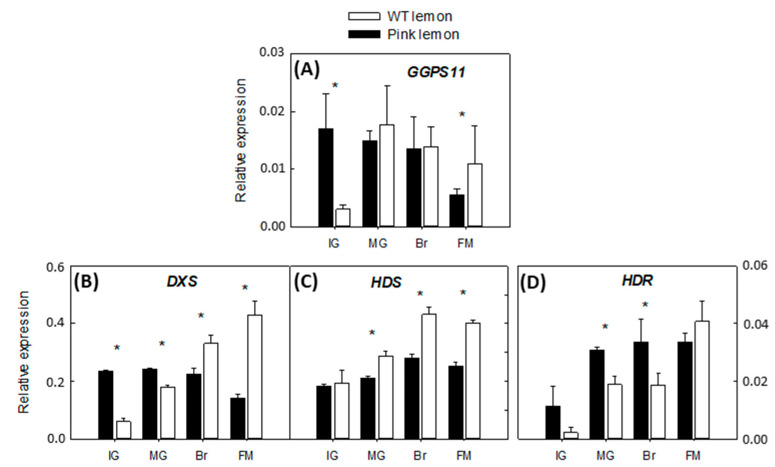
Changes in the expression of genes involved in the MEP pathway: *GGPS11*, geranylgeranyl diphosphate synthase 11 (**A**); *DXS*, 1-deoxy-D-xylulose-5-phosphate synthase (**B**); *HDS,* hydroxymethylbutenyl diphosphate synthase (**C**); *HDR,* hydroxymethylbutenyl diphosphate reductase (**D**), in the pulp of the PL and WT lemon fruit at four developmental stages: IG (immature green), MG (mature green), BR (breaker), FM (fully mature). Asterisks indicate significant differences between genotypes for each developmental stage (*p* < 0.01) by one-way ANOVA (*p* < 0.01).

**Figure 4:**
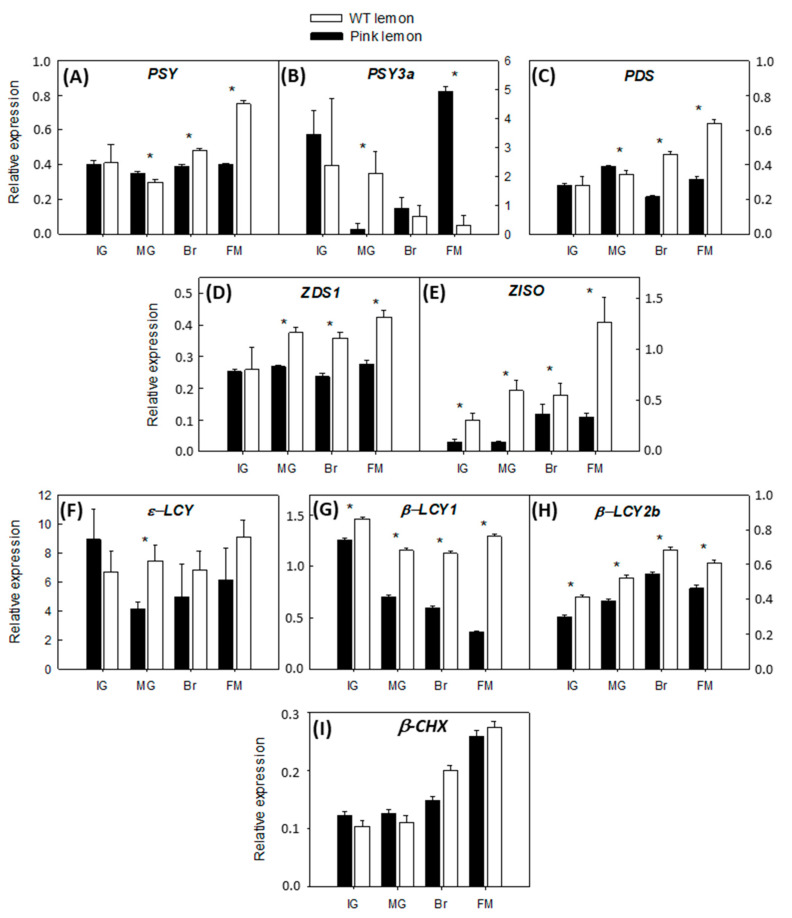
Changes in the expression of genes involved in carotenoids biosynthesis: *PSY*, phytoene synthase (**A**); *PSY3a*, phytoene synthase 3a (**B**); *PDS*, phytoene desaturase (**C**); *ZDS1*, Ƶ-carotene desaturase 1 (**D**); *ZISO*, Ƶ-carotene isomerase (**E**); ε-*LCY*, ε-cyclase (**F**); *β-LCY1*, β-lycopene cyclase 1 (**G**); *β-LCY2b*, β-lycopene cyclase 2b (**H**); *β-CHX*, β-carotene hydroxylase (**I**), in the pulp of PL and WT lemon fruit at four developmental stages: IG (immature green), MG (mature green), BR (breaker), FM (fully mature). Asterisks indicate significant differences between genotypes for each developmental stage (*p* < 0.01) by one-way ANOVA (*p* < 0.01).

**Figure 5:**
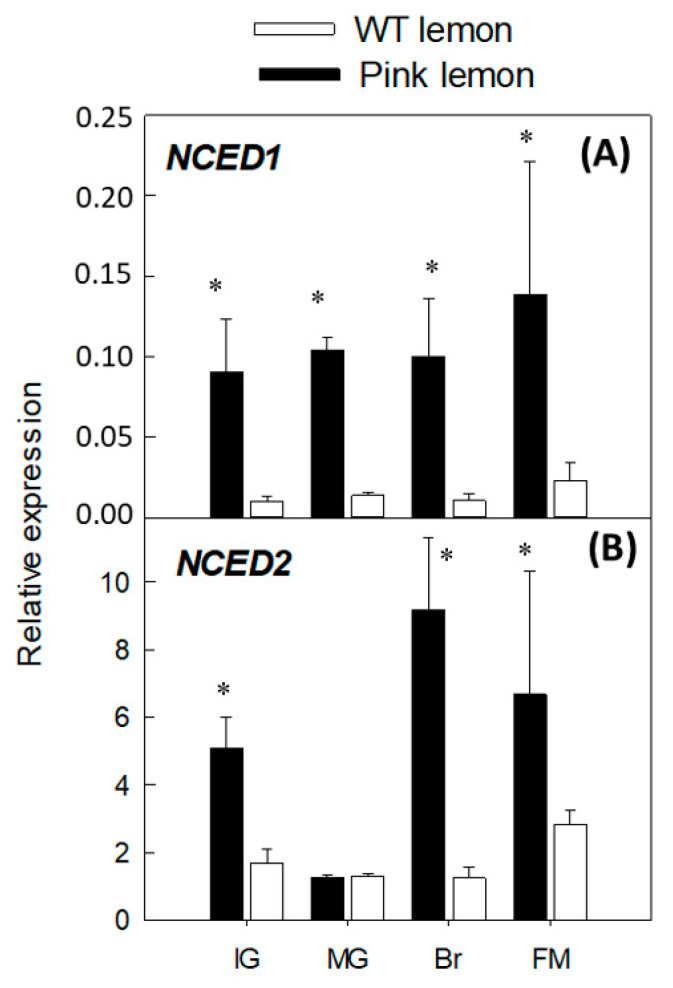
Changes in the expression of genes involved in abscisic acid biosynthesis: *NCED1*, 9-cis-epoxy-carotenoid dioxygenase 1 (**A**); *NCED2*, 9-cis-epoxy-carotenoid dioxygenase 2 (**B**), in the pulp of the PL and WT lemon fruit at four developmental stages: IG (immature green), MG (mature green), BR (breaker), FM (fully mature). Asterisks indicate significant differences between genotypes for each developmental stage (*p* < 0.01) by one-way ANOVA (*p* < 0.01).

**Figure 6:**
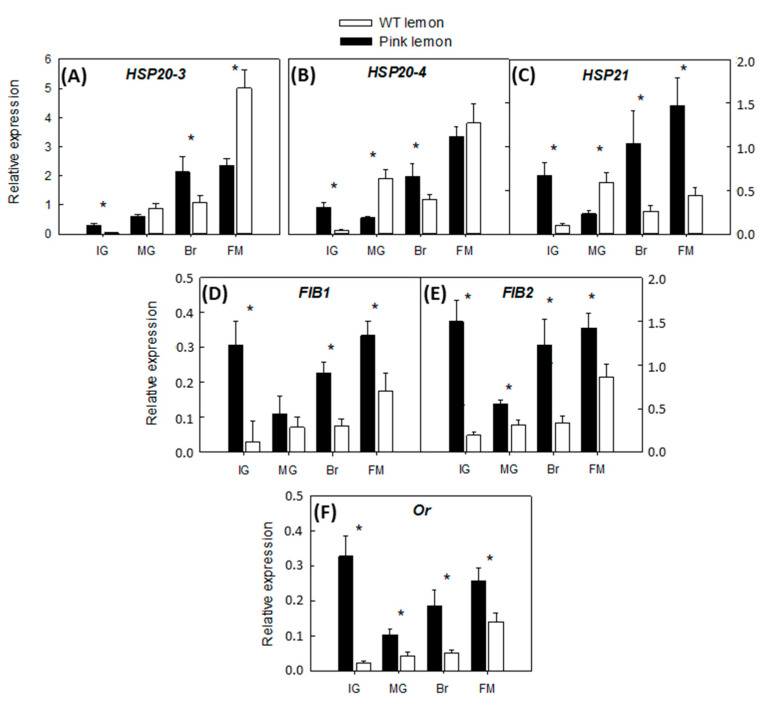
Changes in the expression of genes of carotenoid-associated proteins: *HSP20-3*, heat shock protein 20-3 (**A**); *HSP20-4*, heat shock protein 20-4 (**B**); *HSP21*, heat shock protein 21 (**C**); *FIB1*, fibrillin 1 (**D**); *FIB2*, fibrillin 2 (**E**); *Or*, orange protein gene (**F**), in the pulp of the PL and WT lemon fruit at four developmental stages: IG (immature green), MG (mature green), BR (breaker), FM (fully mature). Asterisks indicate significant differences between genotypes for each developmental stage (*p* < 0.01) by one-way ANOVA (*p* < 0.01).

Differences were highlighted in the expression of several genes on the two genotypes (Figure 3, Figure 4, Figure 5 and Figure 6). In general, the expression of the three genes belonging to the MEP pathway (*DXS*, *HDS* and *HDR*) increased progressively in the pulp of WT. *DXS* and *HDR* were up-regulated at the early development stage in PL compared with WT, while they were down-regulated during the last stages of development. The accumulation of the transcripts corresponding to the plastid-associated *GGPS11* was higher in the pulp of IG mutant lemon and gradually declined during maturation (Figure 3).

Expression of genes involved in early desaturation and isomerization steps of carotenoid biosynthesis, with the exception of *PSY3a*, were up-regulated during maturation in the pulp of WT lemon fruits. Expression of the *PSY*, *PDS*, *ZDS* and *ZISO* genes experienced minor increases in PL mutant fruit and after the MG phase the transcripts accumulation was significantly lower than in WT pulp (Figure 4). The transcription of genes involved in lycopene cyclization also showed important differences between the genotypes under evaluation. The expression of *β-LCY1* remained relatively constant during WT lemon maturation, unlike PL, in which the gene was down-regulated. Although the expression of *β-LCY2* increased in both genotypes during the four ripening stages, it was consistently lower in the pulp of PL mutant than in WT lemon. No significant differences were observed in the accumulation of ε-LCY transcript between the two varieties. *β-CHX* was up-regulated in both WT and PL, following a similar trend (Figure 4).

### 3.4. Expression of Genes Involved in the Biosynthesis of Abscisic Acid

Regarding the genes encoding for 9-cis epoxycarotenoid dioxygenase (*NCED*), which are involved in the production of ABA, both *NCED1* and *NCED2* were up-regulated in WT and PL. However, the level of transcript of *NCED1* and *NCED2* accumulated in PL was from 6 to 9-times and 2.3 to 7-times higher than in WT, respectively (Figure 5).

### 3.5. Expression of Accessory Genes Involved in the Accumulation of Carotenoids

To clarify whether the massive accumulation of carotenoid in the pink-fleshed lemon is associated with alterations in the expression of genes related to chromoplast differentiation and carotenoids-sequestering structures, accumulation of mRNAs corresponding to three *HSP* (*HSP20_3*, *HSP20_4*, *HSP21*), two fibrillins (*FIB1*, *FIB2*) and an *ORANGE* (*OR*) gene was investigated. The relative expression pattern of the *HSP* genes underwent a noticeable up-regulation in WT during the last ripening stage. The most pronounced difference was in the transcription of *HSP21*, which was over-expressed in the pulp of the PL mutant during the developmental stages analyzed. Although the transcription of *FIB1* and *FIB2* genes followed a more constant trend in WT lemon, the level of transcript accumulated in the PL was considerably higher, except for a slight decline at the MG phase. The expression of the *Or* gene followed the same pattern of *FIB1* and *FIB2*. Accumulation of the *Or* transcript was 3.4 to 11-times higher in the PL than the WT lemon (Figure 6).

## 4. Discussion

Although variegated pink-fleshed lemon was identified in 1932 and its fruits are cultivated and purchased in many countries, the biochemical and molecular alteration behind its characteristic pigmentation is still unknown. A detailed observation of the pulp coloration and a comparative analysis of carotenoid content and composition are reported here for the first time, revealing interesting features of this mutant in comparison with other red-fleshed citrus mutants.

The pulp of WT lemon contained negligible amounts of carotenoids at the four developmental stages analyzed (Table 1), in agreement with previous investigations [9,29], reinforcing the classification of lemon as a low-carotenoid accumulating *Citrus* genotype [15]. The red coloration of the pulp was clearly distinguishable in IG fruits (Figure 2A) and lycopene, even at moderated levels (Table 1), was already detectable. These observations indicate that the accumulation of the red pigment was not a ripening-related event like in other red-fleshed citruses like pummelos, grapefruits and oranges mutants. Indeed, lycopene biosynthesis was initiated very early in the fruit development, at the beginning of the second phase of fruit growth during cell enlargement. Accumulation of lycopene in the pulp of PL was associated with a high concentration of phytoene and phytofluene, two colorless carotenes that were virtually absent from the WT lemon (Table 1). The concentration of these two linear carotenes reached a maximum in MG fruits and declined immediately after (Table 1). Other than in the Pinalate orange mutant [46], the accumulation of phytoene and phytofluene is very unusual in lemons and other citrus fruits; in fact, the concentrations found in the PL pulp are among the highest ever reported for citrus fruits [7,47]. HPLC-PDA analysis enabled the detection of small amounts of neurosporene and δ-carotene at early developmental stages; both carotenes are hardly identified in the pulp of citrus fruits [9,47]. This unusual accumulation of δ-carotene, which is a carotene characterized by a ε-ring at one end while the other end is linear, may suggest a defect in the β-cyclization of lycopene that would lead to the accumulation of upstream carotenes, and helps explain the large amounts of phytoene and phytofluene found in the PL (Table 1).

The pattern of carotenoid accumulation in the pulp of PL is different to those found in other lycopene-accumulating citrus fruits. In the case of orange fruits, such as Hong Anliu or Cara Cara [30,31,48,49], and red-pummelos [14,37,38], lycopene is accumulated progressively during maturation and the highest concentration is attained at full maturity. Similarly, the low amount of phytoene accumulated in immature fruits of some mutants gradually increased during maturation [14,30]. In lemon, the expression of genes involved in the biochemical pathway of carotenoid is different to other citrus species [15,29], and non-pigmented lemons have a low capacity to accumulate carotenoids. Then, it is reasonable to assume that the genetic alteration responsible for the accumulation of lycopene in the pink-fleshed lemon may be differ from other citrus mutants characterized by increased levels of lycopene.

The transcriptomic analysis, which included the study of four genes of the MEP pathway (Figure 3) and nine genes of the biosynthesis of carotenoids (Figure 4), highlighted important differences between the two accessions under evaluation, which might clarify the cause of the mutation. In ordinary lemons, the transcription of MEP pathway genes increases with ripening in response to an enhanced demand of precursors due to the formation of downstream products [1,50]. However, in immature PL fruits, the transcription of *GGPS11*, *HDS* and *DXS* was higher than in the WT. These results are in accordance with the extensive differences in total carotenoids content between the two genotypes (20.2 µg/g FW in PL vs. traces in the WT) and may increase the availability of GGPP to be converted into phytoene and other carotenoids. Similar results have been observed in the pulp of the red-fleshed oranges Hong Anliu and Cara Cara, where the accumulation of early carotenes appears to be associated with an increased production of isoprenoid precursors [30,49].

The expression patterns of carotenoid biosynthetic genes in the pulp of WT lemon was similar to the ones characterizing other white-fleshed varieties [36,51]. The low carotenoid content in non-pigmented lemons is generally associated with an up-regulation of the upstream carotenoid genes like *PSY*, *PDS*, *ZDS*, *ZISO, β-LCY2b* and *β-CHX*, while *ε-LCY*, *β-LCY1* are usually down-regulated. In the pulp of PL, however, differences in the pattern of expression of carotenoid biosynthetic genes were not consistent with the alterations in carotenoid content and composition (Figure 4, Table 1). Genes synthesizing the precursors of lycopene were not up-regulated during maturation; on the contrary, the genes related to the β-cyclization of lycopene *β-LCY1* and *β-LCY2b* showed reduced transcript levels in PL than in WT from early stages of development (Figure 4). In particular, this occurred during the major development of the fruit (IG to MG), when carotenoids reached their maximum concentration in the PL (Table 1). In addition, the expression of *β-LCY1* was severely down-regulated in PL relative to WT (Figure 4). Transcripts of *β-LCY2a*, which is the allele with the highest in vitro activity, were not detected in the pulp of both lemon genotypes at any developmental stage (data not shown), indicating a reduced capability of lemon fruits to convert early carotenes to xanthophylls. These alterations, combined with the lower expression of the *β-LCY2b* in PL, may explain the onset of a bottleneck along the carotenoid pathway which could lead to the massive accumulation of lycopene, phytoene and phytofluene (Figure 4). According to this hypothesis, the *PSY* transcription would not be a limiting factor at early developmental stages but it might be more critical during the latter stages. These data are in agreement with those reported for red pummelo, in which the balance between the transcription of genes located upstream and downstream lycopene, together with the reduced LCY activity, led to the accumulation of lycopene [14,36,38]. Our results suggest that *β-CHX* is not a limiting factor for the accumulation of carotenoids in the PL, although the levels of the transcripts are very low compared with other citrus fruits [15,29].

Another key to carotenoid accumulation is the balance between biosynthesis and degradation of metabolites. Indeed, the pool of carotenoids present in the tissues is related to their degradation rate [1,50]. It is reasonable to assume that the remarkable alteration in the carotenoid pool that occurred in the PL could modify the regulatory network operating in ordinary lemons. Thus, besides carotenoid biosynthetic genes, the expression of *NCED1* and *NCED2* were up-regulated in PL relative to WT (Figure 5). These two genes are related to ABA synthesis and operate downstream of xanthophylls production. Therefore, an enhancement in their transcription might suggest an altered homeostasis of the pathway. Although ordinary lemons contain very low amounts of xanthophylls in their flesh, they accumulate considerable quantities of ABA, indicating that the flux of metabolites producing this hormone is active [51,52]. Then, the altered carotenoid composition in the pulp of the PL, likely due to a reduced lycopene cyclization, might de-regulate the normal genetic network of the pathway, causing a positive feedback of the genes involved in ABA formation. These results are similar to those found in the Cara Cara orange mutant, where lycopene accumulation in the flesh was accompanied by a reduction in ABA content and enhanced expression of both *NCED1* and *NCED2* genes [30]. In other plant tissues, it has been shown that alterations in carotenoids composition caused coordinated regulation of *NCED* genes and ABA content [53].

The accumulation of carotenoids in specialized structures is a stable storage system and an alternative mechanism to regulate carotenoids availability [54,55]. The transcriptional analysis of genes encoding for carotenoid-associated proteins highlighted significant alterations in PL. *HPS21*, *FIB1, FIB2* and *OR* genes were consistently over-expressed in the pulp of PL relative to WT (Figure 6). The carotenoid associated-proteins encoded by these three genes were associated with numerous processes involved in carotenoid storage; in addition, they contributed to carotenoid stabilization in plastoglobuli, especially during the transition phase from chloroplast to chromoplast [55]. It has been found that *HSP21* chaperone stimulates accumulation of lycopene in tomato and protects fruit pigmentation from heat-stress, demonstrating its close relationship with carotenoid content [25]. Fibrillin is a family of proteins playing structural functions in the packaging and organization of carotenoids in plant tissues, and constitutes a key element for their storage and metabolism [56]. The carotenoid content and composition in tomato and pepper fruits have been correlated with the transcript abundance of fibrillins [57]. Both *FIB* genes displayed a similar expression pattern during the massive accumulation of carotenoids in the PL, with a high mRNA accumulation at IG and a slight decline at MG (Figure 6). These findings support the involvement of fibrillins in the unusual accumulation of carotenoids in the pulp of the pink-fleshed lemon mutant, where they probably increase the storage capacity of structures that are not usually differentiated in ordinary lemons.

The *OR* gene, first described in cauliflower, enhances carotenoids accumulation and chromoplasts differentiation [34]. OR is considered one of the main post-translation regulator of PSY [58], since recent studies carried out on several plant species found that the OR protein interacts directly with PSY, stabilizing the enzyme and increasing its activity [27,28,59]. Moreover, OR plays a crucial role in the formation of carotenoid-sequestering complexes and the stabilization of carotenoids in plant tissues [58]. The transcription of *Or* gene followed the same pattern of *FIB* in PL (Figure 6), suggesting that both proteins may share critical functions in the stabilization of the large amount of carotenoids accumulated in the mutant. It is tempting to speculate that the overexpression of the *Or* gene in PL may increase PSY stability, enhancing the flow of carotenes into the pathway. In that case, a reduced enzymatic activity or a lower transcription of genes encoding for LYCs would favor the accumulation of lycopene and other upstream carotenes like phytoene and phytofluene in PL. In a transgenic potato tuber overexpressing the *Or* gene, the accumulation of β-carotene was significantly higher than in control samples [60]. On the whole, our results suggest that the over expression of the *OR* gene might be strictly involved in the events connected with the stabilization of the massive amount of carotenoids accumulated by PL. Unfortunately, it is not possible to establish if the up-regulation of carotenoid-associated protein genes was the cause or just the consequence of the lycopene and the others upstream carotenoids accumulation in PL. However, these findings provide novel insights into the metabolic changes that occur in the mutant, which might support further studies aimed at the identification of molecular markers related to the accumulation of lycopene, which is currently one of the most in-demand quality traits in modern fruit crops.

## 5. Conclusions

The carotenoids profile of the pulp of Pink lemon revealed an increased amount of linear carotenes, such as lycopene, phytoene and phytofluene compared to the parental line. Thus, the distinctive phenotype of Pink lemon is associated with an up-regulation and down-regulation of the genes upstream and downstream the lycopene cyclase, respectively. Moreover, accessory genes involved in the accumulation of carotenoids such were overexpressed in the pulp of the pink-fleshed, indicating an enhanced capacity for sequestration, stabilization or accumulation of carotenes. This is the first work where the physiological and molecular characterization of carotenoids accumulation in Pink lemon is reported and provides novel insights into the altered metabolism of carotenoids in this mutant.

## Figures and Tables

**Table 1:** Carotenoid content and composition (µg/g FW) in the pulp of the pink lemon and wild type lemon at four developmental and ripening stages.

	Pink Lemon				Wild Type			
Carotenoids (µg/g FW)	IG	MG	BR	FM	IM	MG	BR	FM
Phytoene	17.59 ± 1.12	44.01 ± 0.90	4.98 ± 0.30	8.81 ± 0.07	tr.	tr.	0.04 ± 0.01	0.04 ± 0.01
Phytofluene	2.27 ± 0.17	8.93 ± 1.75	1.00 ± 0.01	1.79 ± 0.01	nd	nd	nd	tr.
ζ-carotene	nd	0.05 ± 0.01	nd	nd	nd	nd	nd	nd
Neurosporene	0.06 ± 0.01	0.24 ± 0.03	nd	nd	nd	nd	nd	nd
Lycopene	0.24 ± 0.01	0.49 ± 0.13	tr.	0.02±0.01	nd	nd	nd	nd
δ-carotene	tr.	0.04 ± 0.01	nd	nd	nd	nd	nd	nd
Lutein	0.06 ± 0.01	nd	nd	nd	tr.	tr.	tr.	tr.
β-carotene	nd	nd	nd	nd	nd	tr	nd	tr.
β-cryptoxanthin	0.02 ± 0.01	nd	0.02 ± 0.01	nd	nd	0.03 ± 0.01	0.02 ± 0.01	0.02 ± 0.01
Anteraxanthin	nd	nd	nd	nd	nd	nd	tr.	tr.
Violaxanthin	nd	tr	nd	nd	nd	tr.	nd	tr.
Total carotenoids ^α^	20.24 ± 1.54	53.33 ± 2.17	6.01 ± 0.29	10.61 ± 0.03	tr.	0.05 ± 0.01	0.06 ± 0.01	0.08 ± 0.01

The amount of violaxanthin represents the sum of all-trans and 9-cis isomers. Traces indicate amount lower than 0.01 µg/g FW. nd: not detected. Tr: traces. Data are expressed as mean ± SD. ^α^ Total carotenoids are the sum of the main carotenoids identified and quantified.

## Supplementary Materials

The following are available online at https://www.mdpi.com/2073-4425/11/11/1294/s1, Table S1. qRT-PCR primer sequences, Figure S1. HPLC profiles of saponified carotenoid extracts in pulp of fruits of wild type lemon and pink lemon at mature green stage (MG).
