# Supplementary material for: Transcriptional Analysis of Carotenoids Accumulation and Metabolism in a Pink-Fleshed Lemon Mutant
Source: Genes (Basel). 2020 Oct 30;11(11):1294. doi: 10.3390/genes11111294 (PMC7692314; doi:10.3390/genes11111294)
Supplement: Supplementary file 1 [file genes-11-01294-s001.pdf]

**Table S1**. qPCR primer sequences.

| | Gene name | Forward 5'-3' | Reverse 5'-3' | Reference |
|---|---|---|---|---|
| **XM_006464503.3** | ACTINA | TTAACCCCAAGGCCAACAGA | TCCCTCATAGATTGGTACAGTATGAGAC | Alos et al. 2014 |
| **XM_006466273.3** | DXS1 | CGTGTTTTCAACACACCTGACG | AAGCCCCGAAGTCTTCCTCAT | Alos et al. 2006 |
| **XM_006488044.3** | HDS | CTGCCGGAAATTGGACTTCC | CCATCCTGAAGAAGGGTACC | Alquezar et al. 2008 |
| **XM_006487143.3** | HDR1 | AGACCGTGGAATTCCCTCATACG | AGGCACCGGCTGTCACC | Alquezar et al. 2008 |
| **XM_006486607.3** | GGPS11 | CCGAGGTCAGCCCTCAAACC | CTCAGGCACGAGATGGGGG | Lado et al. 2015 |
| **XM_006481880.3** | PSY1 | GGTCGTCCATTTGATATGCTTG | CCTAAGGTCCATCCTCATTCCT | Carmona et al. 2012 |
| **XM_006492653.3** | PSY3a | AATGCATTTTGTGTAAGCCCTGCT | TGTCCCTAAAAGGCTTGATGTGTAAATTG | Manzi et al. 2016 |
| **NM_001288862.1** | PDS | TCCCTTCTAAGTGTGTATGCC | TGCAAGCTCCTTCATTGTAGC | Carmona et al. 2012 |
| **AF372617.1** | ZDS | ACAATCTGTTTGAGGCGCAG | CATAGGTATTGGAAACCCTTACTCC | Carmona et al. 2012 |
| **MG492005.1** | BLCY1 | GAACCAGGAGCTTAGGTCTG | GCTAGGTCTACAACAAGGCC | Carmona et al. 2012 |
| **MG492007.1** | BLCY2a | GAGCAAGTCTCATCGCGTCATAGTG | ACTTTAGCCTTATGAAACTTAACTCCATTTG | Alquezar et al. 2013 |
| **MG492008.1** | BLCY2b | GCAAGTCTCATCGCGTCATGGTA | ACTTTAGCCTTATGAAACCTAACGCCATTTA | Alquezar et al. 2013 |
| **XM_006475429.2** | ε-LCY | AAGGTGTGTCGAGTCAGGTGTTT | CCTGCAGGGGACAATCATATCATGTT | Alquezar et al. 2008 |
| **XM_025102457.1** | BCHX | GGCTCATAAAGCTCTGTGGC | CCAGCACCAAAACAGAGACC | Carmona et al. 2012 |
| **XM_006478830.3** | ZISO | GCAGCGTCACTGGGTTTAAT | GTTCGCCTCTTCACAGCTTC | |
| **AB219179.1** | NCED1 | CCACGATGATAGCTCATCCG | CCACTTGCTGGTCAGGCACC | Rodrigo et al. 2006 |
| **AB219172.1** | NCED2 | CTTCCCAACGAAGTCCATAG | GGATTCCATTGTGATTGCTG | Rodrigo et al. 2006 |
| **XM_006436998.2** | Or | GATGTTGATGTGTTGCGGCGG | AAGTCCTGCACTGTTTCAGGACC | Lado et al. 2015 |
| **AB011797.1** | FIB1 CitPAP | GGTGGCAGAGGAGGAGAG | GGCATTTAGCAGAGTTAAGGC | Lado et al. 2015 |
| **AB011797.1** | FIB2 CitPAP | CCATTGGCGAGGGTGGAGG | CGAACTTGATCTGCACACGCTTG | Lado et al. 2015 |
| **XM_006469901.3** | HSP21 | GGGGAAGAAGAAGAGTGGCC | TGTCGACGATTTTGGCAGTGG | Lado et al. 2015 |
| **XM_006480857.3** | HSP20-3 | ACGTCTGGGCGCCCCTTGG | CTCACCGCTGATCTGAAGGACTC | Lado et al. 2015 |
| **XM_006424900.2** | HSP20-4 | TCCGGTTATTCGCCTGCGC | TGACCGCTTATCTGAAGCACCC | Lado et al. 2015 |

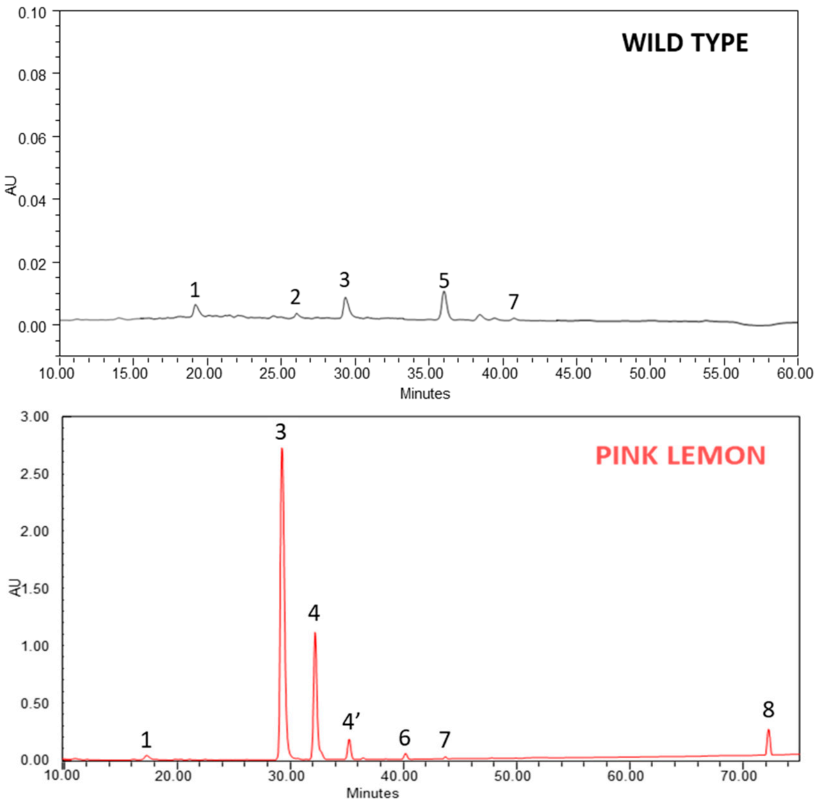


**Figure S1**. HPLC profiles of saponified carotenoid extracts in pulp of fruits of wild type lemon and Pink lemon at mature green stage (MG). All profiles are MaxPlot chromatograms (each carotenoid shown at its individual λ maxima). Note the high concentration of phytoene (peak no. 3), phytofluene isomers (peak no. 4 and 4’) and lycopene (peak no. 8) in Pink lemon extract. AU, Absorption units. The compounds correspond to (1) Violaxanthin; (2) Lutein; (3) Phytoene; (4, 4’) Phytofluene isomers; (5) β-cryptoxanthin; (6) Neurosporene; (7) β-carotene; (8) Lycopene.
